# Solution of the inverse problem for Bessel operator on an interval $[ 1,a ]$

**DOI:** 10.1186/s13660-018-1631-0

**Published:** 2018-02-17

**Authors:** Mesut Coskun, Tuba Gulsen, Hikmet Koyunbakan

**Affiliations:** Department of Mathematics, Faculty of Science, Firat University, Elazig, Turkey

**Keywords:** 34A55, 34L05, 34L20, Inverse nodal problem, Prüfer substitution, Bessel equation

## Abstract

In this note, we solve the inverse nodal problem for Bessel-type *p*-Laplacian problem

$$\begin{aligned}& - \bigl( y^{{\prime} (p-1)} \bigr) ^{\prime} = ( p-1 ) \bigl( \lambda- \omega(x) \bigr) y^{(p-1)},\quad1\leq x\leq a, \\& y(1) =y(a)=0, \end{aligned}$$

on a special interval. We obtain some nodal parameters like nodal points and nodal lengths. In addition, we reconstruct the potential function by nodal points. Results obtained in this paper are similar to the classical Sturm–Liouville problem. However, equations of this type are considered with the condition defined at the origin. We solve the problem on the interval $[1,a]$, that problem is not singular.

## Introduction

By using separation of variables, the wave equation can be written with spherical symmetry

1.1 $$ -y^{{\prime\prime} }(x)+\omega(x)y=\lambda y, $$

where *λ* is a constant referring to the eigenvalue of the problem, and $\omega(x)=\omega_{o}(x)+\frac{l(l+1)}{x^{2}}$ [1], where *l* is a positive integer or zero and $\omega_{o}(x)$ will be defined in what follows.

Let us take into account the eigenvalue problem

1.2 $$\begin{aligned}& - \bigl( y^{{\prime} (p-1)} \bigr) ^{\prime} = ( p-1 ) \bigl( \lambda- \omega(x) \bigr) y^{(p-1)},\quad 1\leq x\leq a, \end{aligned}$$

1.3 $$\begin{aligned}& y(1)=y(a)=0, \end{aligned}$$

where $l=0,1,2,\ldots, p>1$, $a\neq0$, and $y^{(p-1)}= \vert y \vert ^{(p-2)}y$. In this work, we shall assume that $\omega _{0}(x)\in L^{2} [ 1,a ] $ and $y(x,\lambda)$ denotes the solution of problem (1.2)–(1.3). The equation given in (1.1) is taken into account by the condition defined at zero which is singular. So, it is not easy to obtain the solution of the inverse problem. That is why we will consider the problem on the interval $[ 1,a ] $ where the problem is regular. One can consider the singular case of the same problem. Note that for $p=2$, the inverse problem for the Bessel operator has been studied by [2]. In [3], the authors proved that the problem

$$\begin{aligned}& - \bigl( \bigl\vert y^{{\prime} } \bigr\vert ^{(p-2)}y^{{\prime} } \bigr) ^{\prime} = ( p-1 ) \bigl\vert y^{{\prime} } \bigr\vert ^{(p-2)}y, \\& y(0) =0,\qquad y^{{\prime} }(0)=1, \end{aligned}$$

has a solution $S_{p}(x)$, where $S_{p}(x)$ is called the sine function for any *p*, and they also defined inversion of the integral

$$ x= \int_{0}^{S_{p}(x)}\frac{1}{ ( 1-t^{p} ) ^{\frac{1}{p}}}\,dt. $$

Then the first zero $\pi_{p}$ of $S_{p}(x)$ is

$$ \pi_{p}=2 \int_{0}^{1}\frac{1}{ ( 1-t^{p} ) ^{\frac{1}{p}}}\, dt= \frac{2\pi/p}{\sin\pi/p}. $$

Also, the function $S_{p}(x)$ satisfies $\vert S_{p}(x) \vert ^{p}+ \vert S_{p}^{{\prime} }(x) \vert ^{p}=1$, which is similar to the trigonometric identity $\sin^{2}x+\cos^{2}x=1$ for $p=2$.

An inverse nodal problem means finding the potential function through the nodal points (zeros of eigenfunctions) without any other spectral data. Nowadays, solving this problem for one-dimensional *p*-Laplacian problem is more popular. In this problem, given nodal points, one can find the potential function in a general case. At the first stage, Prüfer transformation is significant (see [3–8]). Especially, for $l=0$, we obtain the regular Sturm–Liouville problem, and it has solved by many authors (see [9, 10]).

The zero set $X_{n}= \{ x_{j}^{n} \} _{j=1}^{n-1}$ of the eigenfunction $y_{n}(x)$ corresponding to $\lambda_{n}$ is called the set of nodal points. And $l_{j}^{n}=x_{j+1}^{n}-x_{j}^{n}$ is called the nodal length of $y_{n}$. The eigenfunction $y_{n}(x)$ has exactly $n-1$ nodal points on the interval, say $0=x_{0}^{(n)}< x_{1}^{(n)}<\cdots <x_{n-1}^{(n)}<x_{n}^{(n)}=1$. The inverse nodal problem has been studied, and many reconstructed formulas have been derived and analyzed for different operators by many authors (see [11–16]).

### Lemma 1.1

([17])

- *For* $S_{p}^{\prime} \neq0$,
  $$ \bigl( S_{p}^{\prime} \bigr) ^{\prime} =- \biggl\vert \frac {S_{p}}{S_{p}^{\prime} } \biggr\vert ^{p-2}S_{p}. $$
- $$ \bigl( S_{p}S_{p}^{{\prime} (p-1)} \bigr) ^{\prime} = \bigl\vert S_{p}^{\prime} \bigr\vert ^{p}-(p-1)S_{p}^{p}=1-p \vert S_{p} \vert ^{p}=(1-p)+p \bigl\vert S_{p}^{\prime} \bigr\vert ^{p}. $$

In this study, we peruse the inverse nodal problem of the *p*-Laplacian modified Sturm–Liouville problem with integrable potential on a general interval. Using the modified Prüfer transformation, we will show that the potential function $\omega_{0}(x)$ can be reconstructed by nodal points.

### Results and discussion

In the present paper, we find the potential function by using nodal parameters for the *p*-Laplacian Bessel operator on a regular interval $[1,a]$. Especially, we have used the Prüfer substitution which is also used for regular problems. However, we consider the *p*-Laplacian operator on a regular interval, one can consider it at the origin that the problem is singular. In that time, the results are more interesting.

### Conclusions

We aim to solve an inverse problem for singular operators. For this, by using the Prüfer substitution, we obtain nodal points, nodal length, and the formula for a potential function. We believe that these results will give an idea on the solution of inverse problems for some different singular problems.

### Methods

This paper is organized as follows. In the first section, we give some preliminaries for the Bessel equation and also the properties of nodal parameters. In the second section, we define the Prüfer substitution for a *p*-Laplacian Bessel equation. We also give asymptotic forms of nodal points and nodal lengths. In Section 3, we present a reconstruction formula by nodal lengths for the *p*-Laplacian Bessel operator. The method used in this paper is similar to the method used in the Sturm–Liouville problem.

## Asymptotics of nodal parameters

In this section, we give some properties of (1.2) *p*-Laplacian operator with (1.3) conditions. Let us define the Prüfer transformation for solution *y* of (1.2) as follows:

2.1 $$ \begin{gathered} y(x)=R(x)S_{p} \bigl( \lambda^{1/p} \theta(x,\lambda) \bigr) , \\ y^{\prime} (x)=(l+1)\lambda^{1/p}R(x)S_{p}^{\prime} \bigl( \lambda ^{1/p} \theta(x,\lambda) \bigr) , \end{gathered} $$

or

2.2 $$ \frac{y^{\prime} (x)}{y(x)}=(l+1)\lambda^{1/p}\frac{S_{p}^{\prime} ( \lambda^{1/p} \theta(x,\lambda) )}{S_{p} ( \lambda^{1/p} \theta(x,\lambda) )} , $$

where $R(x)$ is amplitude and $\theta(x)$ is Prüfer variable. By differentiation of (2.2), according to *x* and using Lemma 1.1, we get

2.3 $$ \begin{aligned}[b] \theta^{\prime} (x,\lambda)&= ( l+1 ) + \biggl[ -(l+1)+(l+1)^{1-p}- \frac{(l+1)^{1-p}}{\lambda} \biggl\{ \omega _{o}(x)+\frac{l(l+1)}{x^{2}} \biggr\} \biggr]\\ &\quad {} \times S_{p}^{p} \bigl( \lambda^{1/p} \theta(x,\lambda ) \bigr) . \end{aligned} $$

### Lemma 2.1

([5])

*Consider* $\theta(x,\lambda _{n})$ *as in* (2.1) *and* $\phi_{n}(x)=S_{p}^{p} ( \lambda_{n}^{1/p} \theta(x,\lambda_{n}) ) -\frac{1}{p}$. *Then*, *for any* $g\in L^{1}(1,a)$,

$$ \int_{1}^{a}\phi_{n}(x)g(x)\,dx=0, $$

*which is known as the generalized Riemann–Lebesgue lemma*.

Now, we can give eigenvalues and nodal parameters for problem (1.2), (1.3).

### Theorem 2.1

*For the problem given in* (1.2), (1.3),

$$ \lambda_{n}^{1/p}=\frac{n\pi_{p}}{\tilde{l} ( a-1 )} +\frac {(l+1)^{1-p}\tilde{l} ^{p-2}(a-1)^{p-2}}{p(n\pi_{p})^{p-1}} \int _{1}^{a} \biggl\{ \omega_{o}(s)+ \frac{l(l+1)}{s^{2}} \biggr\} \, ds+O \biggl( \frac{1}{n^{p-1}} \biggr) , $$

*as* $n\rightarrow\infty$, *where* $\tilde{l} =(l+1) ( 1-\frac {1}{p}+\frac{1}{p(l+1)^{p}} ) $.

### Proof

For problem (1.2)–(1.3), let $\theta(1)=0$. Integrating (2.3) from 1 to *a*

$$\begin{aligned} \theta(a,\lambda) =&(l+1) (a-1) \\ &{}+ \int_{1}^{a} \biggl[ -(l+1)+(l+1)^{1-p}- \frac{(l+1)^{1-p}\omega (x)}{\lambda} \biggr] S_{p}^{p} \bigl( \lambda^{1/p} \theta(x,\lambda ) \bigr) \,dx. \end{aligned}$$

Let $\lambda_{n}$ be an eigenvalue. By Lemma 2.1, we know that

$$ \int_{1}^{a}\rho(x) \biggl\{ S_{p}^{p} \bigl( \lambda_{n}^{1/p} \theta (x,\lambda_{n}) \bigr) -\frac{1}{p} \biggr\} \,dx=o(1),\quad\text{as } n\rightarrow\infty, $$

where $\rho(x)$ is continuous on $[ 1,a ] $. Hence

2.4 $$ \theta(a,\lambda_{n})=\tilde{l} (a-1)-\frac{(l+1)^{1-p}}{p\lambda _{n}} \int_{1}^{a}\omega(s)\,ds+O \biggl( \frac{1}{\lambda_{n}} \biggr) . $$

On the other hand, letting $\theta(a,\lambda_{n})=\frac{n\pi _{p}}{\lambda_{n}^{1/p}}$, we get

2.5 $$ \frac{1}{\lambda_{n}^{1/p}}=\frac{\tilde{l} (a-1)}{n\pi_{p}}-\frac{ (l+1)^{1-p}\tilde{l} ^{p}(a-1)^{p}}{p ( n\pi_{p} ) ^{p+1}} \int_{1}^{a}\omega(s)\,ds+O \biggl( \frac{1}{n^{p+1}} \biggr) , $$

and thus

$$ \lambda_{n}^{1/p}=\frac{n\pi_{p}}{\tilde{l} ( a-1 ) }+\frac {(l+1)^{1-p}\tilde{l} ^{p-2}(a-1)^{p-2}}{p(n\pi_{p})^{p-1}} \int _{1}^{a}\omega(s)\,ds+O \biggl( \frac{1}{n^{p-1}} \biggr) . $$

This completes the proof. □

### Theorem 2.2

*Nodal points of problem* (1.2), (1.3) *have the form*

2.6 $$ \begin{aligned}[b] x_{j}^{n}&=1+ \frac{j(a-1)\tilde{l} }{(l+1)n}-\frac{j\tilde{l} ^{p}(a-1)^{p}}{p(l+1)^{p}n^{p+1}\pi_{p}^{p}} \int_{1}^{a}\omega(s)\, ds \\ &\quad{}+ \int_{1}^{x_{j}^{n}}S_{p}^{p}\,ds- \frac{1}{(l+1)^{p}} \int _{1}^{x_{j}^{n}} \biggl\{ 1-\frac{\tilde{l}^{p}(a-1)^{p}w(s)}{(n\pi _{p})^{p}} \biggr\} S_{p}^{p}\,ds+O \biggl( \frac{j}{n^{p+1}} \biggr) \end{aligned} $$

*as* $n\rightarrow\infty$.

### Proof

Integrating (2.3) from 1 to $x_{j}^{n}$, we get

$$ \frac{j\pi_{p}}{\lambda_{n}^{1/p}}=(l+1) \bigl(x_{j}^{n}-1\bigr)-(l+1) \int _{1}^{x_{j}^{n}}S_{p}^{p} \,ds+(l+1)^{1-p} \int _{1}^{x_{j}^{n}} \biggl\{ 1-\frac{w(s)}{\lambda_{n}} \biggr\} S_{p}^{p}\,ds. $$

By considering the asymptotic estimates of eigenvalues, we obtain (2.6). □

### Theorem 2.3

*The nodal lengths of problem* (1.2), (1.3) *are*

2.7 $$ \begin{aligned}[b] l_{j}^{n}&= \frac{\tilde{l}(a-1) }{n(l+1)}-\frac{\tilde{l} ^{p}(a-1)^{p}}{p(l+1)^{p}n^{p+1}\pi_{p}^{p}} \int_{1}^{a}\omega(s)\,ds \\ &\quad{}+ \int_{x_{j}^{n}}^{x_{j+1}^{n}}S_{p}^{p}\,ds- \frac {1}{(l+1)^{p}} \int _{x_{j}^{n}}^{x_{j+1}^{n}} \biggl\{ 1-\frac{\tilde{l} ^{p}(a-1)^{p}}{ ( n\pi_{p} ) ^{p}} \omega(s) \biggr\} S_{p}^{p}\,ds +O \biggl( \frac{1}{n^{p+1}} \biggr) . \end{aligned} $$

### Proof

When we integrate (2.3) on $[x_{j}^{n},x_{j+1}^{n}]$ and take into account the definition of nodal lengths, we get

$$ \frac{\pi_{p}}{\lambda _{n}^{1/p}}=(l+1) \bigl(x_{j+1}^{n}-x_{j}^{n} \bigr)-(l+1) \int _{x_{j}^{n}}^{x_{j+1}^{n}}S_{p}^{p} \,ds+(l+1)^{1-p} \int _{x_{j}^{n}}^{x_{j+1}^{n}} \biggl\{ 1-\frac{\omega(s)}{\lambda_{n}} \biggr\} S_{p}^{p}\,ds, $$

and formula (2.7) can be easily obtained. □

## Reconstruction of the potential function in *p*-Laplacian Bessel equation

In this part, we prove Theorem 3.1, which means a formula by nodal lengths. Finally, we show that there is a function $F_{n}(x)$ converging to $\omega(x) $ for $n\rightarrow\infty$. However, the method used in this part is similar to the regular boundary value problem, we consider *p*-Laplacian Bessel equation on a general interval as $[1,a]$ (see [4, 18, 19]).

### Theorem 3.1

*Let* $\omega(x)\in L^{2} [ 1,a ] $. *Then*

3.1 $$ \omega(x)=\lim_{n\rightarrow\infty}p(l+1)^{p-1}\lambda_{n} \biggl( \frac{\tilde{l} \lambda_{n}^{\frac{1}{p}}}{\pi_{p}}l_{j}^{n}-1 \biggr), $$

*for* $j=j_{n}(x)=\max \{ j:x_{j}^{n}< x \} $.

### Proof

We need to consider Theorem 2.3 to derive the reconstructed formula for the potential function. After some straightforward computations, we have

$$\begin{aligned} l_{j}^{n} =&\frac{\pi_{p}}{(l+1)\lambda_{n}^{1/p}}+\frac{1}{p} \int _{x_{j}^{n}}^{x_{j+1}^{n}}\,ds-\frac{1}{p(l+1)^{p}} \int _{x_{j}^{n}}^{x_{j+1}^{n}} \biggl( 1-\frac{\omega(s)}{\lambda_{n}} \biggr) \,ds \\ &{}+ \int_{x_{j}^{n}}^{x_{j+1}^{n}} \biggl( S_{p}^{p}- \frac{1}{p} \biggr) \,ds-\frac{1}{(l+1)^{p}} \int_{x_{j}^{n}}^{x_{j+1}^{n}} \biggl( 1-\frac {\omega(s)}{\lambda_{n}} \biggr) \biggl( S_{p}^{p}-\frac{1}{p} \biggr) \,ds. \end{aligned}$$

Furthermore,

$$\begin{aligned} \frac{p(l+1)^{p}\lambda_{n}^{\frac{1}{p}+1}}{\pi_{p}}l_{j}^{n} &=p(l+1)^{p-1} \lambda_{n}+\frac{ ( (l+1)^{p}-1 ) \lambda _{n}^{\frac{1}{p}+1}}{\pi_{p}}l_{j}^{n} \\ &\quad {}+\frac{\lambda_{n}^{\frac{1}{p}}}{\pi_{p}} \int _{x_{j}^{n}}^{x_{j+1}^{n}}\omega(s)\,ds+\frac{p(l+1)^{p}\lambda_{n}^{ \frac{1}{p}+1}}{\pi_{p}} \int_{x_{j}^{n}}^{x_{j+1}^{n}} \biggl( S_{p}^{p}- \frac{1}{p} \biggr) \,ds \\ &\quad {}-\frac{p\lambda_{n}^{\frac{1}{p}+1}}{\pi_{p}} \int _{x_{j}^{n}}^{x_{j+1}^{n}} \biggl( 1-\frac{\omega(s)}{\lambda_{n}} \biggr) \biggl( S_{p}^{p}-\frac{1}{p} \biggr) \,ds. \end{aligned} $$

Then, by using a similar way as in [5], for $j=j_{n}(x)=\max \{ j:x_{j}^{n}< x \} $, we have

$$ \frac{\lambda_{n}^{1/p}}{\pi_{p}} \int _{x_{j}^{n}}^{x_{j+1}^{n}}\omega(s)\,ds\rightarrow\omega(x), $$

and

$$\begin{aligned}& \frac{p(l+1)^{p}\lambda_{n}^{\frac{1}{p}+1}}{\pi_{p}} \int _{x_{j}^{n}}^{x_{j+1}^{n}} \biggl( S_{p}^{p}- \frac{1}{p} \biggr) \,ds \rightarrow0, \\& \frac{p\lambda_{n}^{\frac{1}{p}+1}}{\pi_{p}} \int _{x_{j}^{n}}^{x_{j+1}^{n}} \biggl( 1-\frac{\omega(s)}{\lambda_{n}} \biggr) \biggl( S_{p}^{p}-\frac{1}{p} \biggr) \,ds \rightarrow0 \end{aligned}$$

pointwise converge almost everywhere. Hence, we get

$$ \omega(x)=\lim_{n\rightarrow\infty}p(l+1)^{p-1}\lambda_{n} \biggl( \frac{\tilde{l} \lambda_{n}^{\frac{1}{p}}}{\pi_{p}}l_{j}^{n}-1 \biggr) . $$

 □

### Theorem 3.2

*Let* $\{ l_{j}^{(n)}:j=1,2,\ldots,n-1 \} _{n=2}^{\infty}$ *be a set of nodal lengths of* (1.2)*–*(1.3), *where* $\omega\in L^{2} [ 1,a ] $. *Furthermore*, *let us define*

3.2 $$ F_{n}(x)=\frac{p(l+1)^{p-1} ( n\pi_{p} ) ^{p}}{\tilde {l}^{p}(a-1)^{p}} \biggl( \frac{n l_{j}^{(n)}}{a-1}-1 \biggr) + \frac {1}{\tilde{l} ( a-1 ) } \int_{1}^{a}\omega(s)\,ds. $$

*Then* $\{F_{n}(x)\}$ *converges to* *ω* *almost everywhere in* $L^{1}(1,a)$.

### Proof

By Theorem 3.2, we achieve

$$\begin{aligned} p(l+1)^{p-1}\lambda_{n} \biggl( \frac{\tilde{l} \lambda_{n}^{\frac {1}{p}}}{\pi_{p}}l_{j}^{n}-1 \biggr) =&p(l+1)^{p-1}\lambda_{n} \biggl( \frac{n l_{j}^{(n)}}{a-1}-1 \biggr) \\ &{}+\frac{nl_{j}^{n}}{\tilde{l}(a-1)^{2}} \int_{1}^{a}\omega (s)\,ds+o(1). \end{aligned}$$

Considering $n l_{j}^{(n)}=a-1+o(1)$, as $n\rightarrow\infty$, this implies that

$$ \frac{p(l+1)^{p-1} ( n\pi_{p} ) ^{p}}{\tilde {l}^{p}(a-1)^{p}} \biggl( \frac{n l_{j}^{(n)}}{a-1}-1 \biggr) \rightarrow \omega(x)- \frac{1}{\tilde{l} ( a-1 ) } \int_{1}^{a}\omega (s)\,ds $$

pointwise converges almost everywhere in $L^{1}(1,a)$. □
